# Social capital as a protective resource in times of social crisis—lessons learned from the COVID-19 pandemic. A mixed-method study protocol

**DOI:** 10.3389/fpubh.2025.1648074

**Published:** 2025-10-20

**Authors:** Malin Eriksson, Ailiana Santosa, Liv Zetterberg, Simone Scarpa, Nawi Ng

**Affiliations:** ^1^Department of Social Work, Umeå University, Umeå, Sweden; ^2^School of Social Science, Södertörn University, Stockholm, Sweden; ^3^School of Public Health and Community Medicine, University of Gothenburg, Gothenburg, Sweden

**Keywords:** social capital, COVID-19, mixed methods, Northern Sweden, social crisis

## Abstract

The COVID-19 pandemic caused a global health crisis that affected every aspect of society worldwide. However, the detrimental health effects of the pandemic were not equally distributed across groups and places. Likewise, adherence to preventive measures varied across groups and communities. Having supportive social networks and living in areas with social cohesion—social capital—is believed to protect against adverse consequences of social crises. This mixed method study aims to investigate the bidirectional relationship between social capital and the COVID-19 pandemic, and to analyze the significance of individual and neighborhood social capital for physical and mental health, attitudes toward- and adherence to preventive measures. The specific objectives are to; (1) Investigate the development of neighborhood social capital during the COVID-19 pandemic and to assess whether perceptions on how the pandemic affected life situation and attitudes toward preventive measures differ across neighborhoods with different social capital profiles and population characteristics. (2) Analyze the effects of individual social capital on physical and mental health, as well as attitudes toward and adherence to preventive measures for diverse population sub-groups, living in neighborhoods with different social capital profiles. (3) Analyze how the pandemic and its associated preventive measures impacted people’s access to and utilization of social capital. Sub-study 1 will utilize data from repeated cross-sectional social capital surveys conducted before and after the COVID-19 pandemic, in 2020 and 2024. Data from a cohort who responded to both the post- and the pre-COVID social capital surveys will be linked to population register data on socioeconomic and sociodemographic factors and health registers to be used for the quantitative sub-study 2. A strategic sample of individuals who participated in both the 2020 and the 2024 social capital surveys will be invited to participate in interviews for a subsequent qualitative sub-study 3. This study is carried out in Umeå Municipality, Northern Sweden, where extensive research on social capital, health and social sustainability has been conducted since 2006. The proposed study contributes novel knowledge on how a social crisis affects unequal living conditions between groups and places. This knowledge can provide a basis for what actions are needed to reduce adverse health consequences of social crises.

## Introduction

The COVID-19 pandemic caused a global health crisis that affected every aspect of society worldwide. However, the detrimental effects of the pandemic on physical and mental health were not equally distributed across social groups and places (1). Likewise, adherence to preventive measures, such as vaccination, varied across groups and communities. It is well established that existing social inequalities are reinforced during societal crises, with vulnerable groups experiencing the most far-reaching consequences (2). The protective role of social networks for health has been recognized for a long time (3). Having supportive social networks and living in an area with strong social cohesion—i.e., having access to social capital—is believed to protect against the adverse health consequences of social crises. However, social distancing and self-isolation measures during the pandemic reduced opportunities for social interaction simultaneous as the need for social support increased. Social capital has proven to be a relatively stable resource during times of social stability, but more likely to fluctuate in times of social crisis (4). Thus, the COVID-19 pandemic may have influenced both the availability and the use of social capital at individual and community levels.

Social capital is conceptualized as both an individual and a collective (place-specific) feature and concerns “social networks, the reciprocities that arise from them and the value of these for achieving mutual goals” (5). Hence, social capital is a multifaceted concept encompassing social interactions and group membership that can affect well-being at individual and community levels. The concept has gained considerable attraction in research and policy over the past decades, including studies on resilience and crisis management (6, 7) and public health (8). This makes social capital an appealing framework to understand responses to societal crises.

The individual (social network) approach is rooted in sociology and defines social capital as “*the ability of actors to secure benefits by membership in social networks or other social structures*” (9). The underlying idea is that individuals can access valuable resources, such as social support and information, through their social networks. These resources can become particularly salient in times of crisis. Moreover, social networks may also provide access to role models who influence health behavior and adherence to public health initiatives such as vaccination (10). Further, participation in social networks characterized by reciprocity norms may also increase trust not only within the networks themselves but also toward public institutions. The concepts of bonding and bridging social capital are useful for exploring which forms of social capital may be mobilized during a social crisis and by whom. Bonding social capital refers to strong ties within a network of people who share similar characteristics, e.g., ethnicity or socioeconomic position, while bridging social capital refers to weaker ties that link individuals from more diverse and heterogeneous networks (11). Access to these forms of social capital is often unequally distributed. According to Bourdieu (12), inclusion in social networks depends on individual “investment strategies,” whereby those with greater resources are more likely to access influential bridging networks. In contrast, vulnerable groups tend to have poorer social networks and, therefore, fewer social capital resources to draw in times of crisis.

The collective (social cohesion) approach views social capital as a property of places characterized by levels of social participation, generalized trust, and reciprocity norms (11). In this framework, social capital is conceptualized in terms of resources available to individuals and groups because of social cohesion, e.g., the ability of communities to undertake collective action, or the presence of norms of mutual help and support (11). This approach builds mainly on the work of the political scientist Robert Putnam (13, 14). He asserted that communities with dense and robust social networks, high participation in these networks, and widespread trust are those with high social capital levels. Residents in such places are more likely to care for and support each other, which could be particularly important in times of social crisis. A key assumption is that place-specific social capital has spillover effects in that living in such a place could benefit all, even individuals who are not socially engaged. Non-engaged individuals can still benefit as their neighbors engage and care for the local community, facilitating the spread of information and resources within the neighborhood. This makes place-specific social capital especially relevant for crisis management and public health interventions, as it suggests that area-based interventions may benefit the entire population, including those with limited social networks. However, research also indicates the risk of social exclusion and decline in trust if negative bonding social capital is developed at the expense of bridging social capital (15, 16). The same mechanisms that spread healthy norms in a community may also lead to social exclusion of groups that do not manage to conform to the norms.

Hence, social capital could potentially affect how individuals and communities manage crises in multiple ways. At the individual level, social capital can ensure access to support and information, even in periods of social restrictions. At the community level, social capital can preserve valuable resources, such as trust, support, spread of norms and information, and collective action. A UK study among older people found that decreased access to local public support services during COVID-19 were associated with poorer mental health (17). Various studies have found protective health effects from social networks (18–21) as well as community-level social capital (22) during the COVID-19 pandemic. Likewise, studies have found that neighborhoods with low levels of social capital experienced worse health outcomes during the COVID-19 pandemic (23, 24). During previous influenza pandemics, different forms of social capital were associated with health-protective behaviors (25), and during the AH1N1 pandemic, neighborhood social capital was found to be associated with an increased likelihood of vaccinating children (26). In addition, studies have found that societies and neighborhoods with high levels of social capital tend to be more resilient, recover more quickly, and facilitate people’s adaptation to changing environments during extraordinary times, including the COVID-19 pandemic (7, 27, 28). A recent study from Japan additionally found that adherence to preventive measures during later stages of the COVID-19 pandemic reduced in areas with low levels of social capital, while not in areas with high levels of social capital (29). Hence, strong social capital at individual and community levels may be an essential resource in social crises through mobilizing and sharing resources, facilitating policy compliance, and for recovering in post-disaster communities. However, face-to-face interactions may also facilitate viral transmission. Therefore, social capital may simultaneously mitigate and exacerbate health risks during a pandemic (30).

Pandemic-related measures such as social distancing and self-isolation reduced opportunities for social interaction and may have limited individuals’ ability to access social support from their broader networks. Consequently, close social networks might have become even more important during the pandemic. A study from Egypt about the impact of the COVID-19 pandemic on mental health and social support found that social support from the closest circle of family members increased more than that from friends during the pandemic, indicating the importance of proximity during times of social crisis (31). For people without close family networks, contact with neighbors may have been essential for accessing information and support. A recent literature review additionally indicates that access to social networks decreases during times of crisis, especially for older and marginalized groups (32).

Place-specific social capital is believed to be relatively stable in times of social stability while more likely to fluctuate in times of social crisis (4). A UK study found that the perceived levels of social cohesion were lower in June 2020 compared to pre-pandemic periods, with the sharpest decline in the most deprived communities, among certain ethnic minority groups and among individuals in the lower-skilled occupations (33). Therefore, in addition to examining the potential protective role of social capital during a pandemic, it is also essential to consider how individual and place-specific social capital interacts—and how this interaction may have shaped pandemic experiences within and across neighborhoods. The pandemic has affected different population groups in divergent ways, even those living in the same neighborhoods, depending on various factors (such as age, prior health status, occupation, and household structure). For instance, older people living independently at home or those experiencing financial hardship due to loss of employment particularly needed support from their social networks. Further, individuals forced to work from home, and families forced to keep children home from school were more likely to suffer from social isolation. In contrast, people with occupations perceived as societal essential functions were forced to go to work and thus more susceptible to infection. These different forms of vulnerability also cut across different socioeconomic groups. An underexplored question is whether the COVID-19 pandemic altered the dynamics of individual and neighborhood social capital, and to what extent social capital influenced individuals’ well-being and their adherence to preventive measures during the pandemic. To date, most research on social capital has relied on cross-sectional data, limiting the ability to draw causal inferences. A key methodological challenge in examining the effects of social capital is the absence of baseline measurements. This study addresses that challenge by leveraging data collected on social capital levels in 46 neighborhoods both before and after the COVID-19 pandemic.

This study aims to investigate the bidirectional relationship between social capital and the COVID-19 pandemic, and to analyze the significance of individual and neighborhood social capital for physical and mental health, attitudes toward- and adherence to preventive measures.

The specific objectives are to:

1 Investigate the development of neighborhood social capital during the COVID-19 pandemic and to assess whether perceptions on how the pandemic affected life situation and attitudes toward preventive measures differ across neighborhoods with different social capital profiles and population characteristics.
2 Analyze the effects of individual social capital on physical and mental health, as well as attitudes toward and adherence to preventive measures for diverse population sub-groups, living in neighborhoods with different social capital profiles.
3 Analyze how the pandemic and its associated preventive measures impacted people’s access to and utilization of individual social capital.

## Methods and analysis

### Study context

This study is conducted in Umeå Municipality, Northern Sweden, and builds on our extensive previous research on social capital, health, and social sustainability in the region. A population-based survey on social capital and health was first distributed in 2006, followed by a second survey in early 2020, just prior to the initial COVID-19 outbreak in Sweden. A third survey was conducted in 2024, capturing post-pandemic conditions. These longitudinal data make Umeå Municipality a particularly compelling case, offering a unique opportunity to examine the role of social capital during the pandemic and to analyze the unequal health impacts across social groups and residential areas.

Umeå municipality is growing and has a vision to reach 200,000 inhabitants by 2050 while maintaining social, ecological, cultural, and economic sustainability. The municipality has long adopted a strategic approach to social sustainability, exemplified by the establishment of the Commission for a Socially Sustainable Umeå in 2017. Our longitudinal research is conducted in close collaboration with this Commission, and our previous studies have made several key contributions to the study of social capital and health in Umeå Municipality. First, we have developed and validated an instrument for measuring social capital at both individual and neighborhood levels (34). Second, we have mapped social capital levels in 46 neighborhoods at three time points: 2006, 2020, and 2024 (35, 36). Third, we have examined associations between individual- and neighborhood-level social capital and various health outcomes (35, 37). Fourth, we have conducted qualitative follow-up studies demonstrating that neighborhood social capital significantly influences resident’s perceived health, and that physical and social environments mutually reinforce one another (38). Fifth, we have conducted register-based analyses revealing that neighborhood social capital can have protective health effects, such as reducing injury risk among young girls (39). Further, we have explored children’s perceptions of health-promoting environments, highlighting how access to community spaces that facilitate children’s social interaction is unevenly distributed across neighborhoods (40).

Our follow-up social capital survey in 2020 showed that neighborhood social capital remained relatively stable over 14 years in the municipality. However, certain demographic characteristics, such as higher proportions of pensioners and families with children and a lower proportion of foreign-born residents were associated with higher levels of social capital (36). A pilot telephone survey conducted during the first phase of the COVID-19 pandemic in Sweden, among a sub-sample of participants from the 2006 survey, indicated increased social interaction and perceived social support in neighborhoods with high levels of social capital. Residents of these neighborhoods more frequently reported improved health during the ongoing pandemic compared to residents of low-social capital neighborhoods (41).

### Study design

This study builds on a mixed-method study design, implying that we combine quantitative and qualitative research methods. We will use a priority sequence model that relies on the complementary principle (42), in that we will follow up quantitative results with subsequent qualitative studies (43). As previously noted, a baseline social capital survey was distributed in 2006 to residents in 46 neighborhoods within Umeå Municipality. The current study builds on this foundation by utilizing data from a pre-COVID social capital survey conducted in spring 2020, as well as data from a post-COVID survey carried out in spring 2024 in the same neighborhoods. In addition, a qualitative follow-up study will be conducted to yield a deeper understanding of the survey results on the role of social capital during the pandemic for different social groups and neighborhoods.

The repeated cross-sectional surveys were distributed to a representative sample of adults aged 18–84 years living in 46 defined neighborhoods within Umeå Municipality. Data from both surveys will be used in the quantitative sub-study 1 of the project. In conjunction with the repeated cross-sectional study, the cohort who participated in the 2020 survey (*N* = 4,947) was also invited to take part in the 2024 survey. This follow-up was made possible through Statistics Sweden, which securely stored the contact information of the 2020 respondents in accordance with ethical approval granted by the Swedish Ethical Authority (Dnr: 202-00160). The follow-up response rate among this cohort was 70%, consistent with previous studies (41). To minimize attrition, Statistics Sweden (SCB) sent three reminders during data collection. We will assess potential attrition bias by comparing baseline characteristics of participants who remain in the cohort with those lost to follow-up. Missing data will be addressed analytically using multiple imputation and mixed-effects models, which are robust under missing-at-random assumptions. We will also conduct a sensitivity analysis to assess if our results change under different missing-not-at-random (MNAR) assumptions. Although some residual risk of attrition bias may remain, these steps will reduce its potential impact on study findings. Data from this cohort will be linked to population register data on socioeconomic and sociodemographic factors, as well as health registers, and will be used for the quantitative sub-study 2 in this study. Next, a strategic sample of individuals who participated in both the 2020 and the 2024 social capital surveys will be invited to participate in interviews for the qualitative sub-study 3 of the project.

Our social capital survey instrument, used in 2006, 2020 and 2024 surveys, measures neighborhood and individual-level social capital. The baseline instrument for social capital (2006) was developed based on a review of existing literature and adapted to a Northern Sweden context (34). The survey instrument covers questions on socioeconomic and background factors, perceptions about living areas, civic and political engagement, reciprocity and trust, social networks, social support, and self-rated health. In 2020, we added questions on physical and mental health and health-related quality of life. In the post-COVID survey in 2024, we included questions on perceptions on how the pandemic affected life situation (health, social, economic and work), and attitudes toward and adherence to COVID-19 preventive measures. In addition, survey participants were asked if they were interested in participating in follow-up interviews by voluntarily providing their contact information in the survey.

Confounders will be identified *a priori* based on theoretical frameworks, prior research, and, where applicable, directed acyclic graphs. In our analyses, a consistent core set of sociodemographic and health-related variables will be adjusted for across models. To reduce the risk of false-positive findings due to multiple testing, we will predefine primary exposures and outcomes, clearly label exploratory analyses, and interpret results with attention to effect sizes, confidence intervals, and consistency across analyses rather than on statistical significance alone.

The three sub-studies are described in detail below.

1 Quantitative sub-study 1: development of neighborhood social capital and variations in perceptions on how the pandemic affected life situation, and attitudes toward COVID-19 preventive measures

This sub-study will address our first specific aim: *to investigate the development of neighborhood social capital during the COVID-19 pandemic* (i.e., *in 2020 and 2024*) *and to assess whether perceptions regarding the pandemic’s impact on life situation, and attitudes toward preventive measures,* var*y across neighborhoods with different social capital profiles and population characteristics.*

This ecological sub-study will be based on data from cross-sectional social capital surveys in 46 neighborhoods in 2020 and 2024, with response rate of 37 and 34%, respectively. Survey weights provided by Statistics Sweden will be applied to account for sampling design and non-response, and multiple imputation techniques will be used to address missing data.

We operationalize neighborhood social capital using five indicators reflecting individual’s perceptions of the social climate in their local neighborhoods, and three indicators of conventional social capital, i.e., social participation, trust and voting, measured by the following questions:

i) “Is it common in this neighborhood that neighbors talk to each other?” (Yes, very common; Yes, rather common; No, rather uncommon; No, very uncommon; No opinion)
ii) “In my neighborhood, people are ready to help each other.” (About enough; Too much; Too little; No opinion)
iii) “In my neighborhood, one is expected to be involved in issues that concern this place.” (About enough; Too much; Too little; No opinion)
iv) “In my neighborhood, people care for each other.” (About enough; Too much; Too little; No opinion).
v) “Did you vote in the last election?” (Yes; No)
vi) “During the last 12 months, have you participated in any social events?” (Yes; No)
vii) “Do you feel that you can trust people in general?” (Yes; No)

We will construct a multidimensional index of neighborhood social capital from these seven variables using Multiple Correspondence Analysis (MCA). The analysis will begin with descriptive statistics and visualizations (such as frequency distributions and bar charts) of these variables to ensure transparency. We will then examine each variable’s contributions to the MCA dimensions and assess the proportion of variance explained to ensure the robustness of our analysis. Following the procedure outlined in our previous work (35, 36), we will average the individual-level index scores to the neighborhood-level. Finally, neighborhoods will be ranked and categorized into social capital quintiles, from very low to very high.

In the analysis, neighborhoods will be grouped according to changes in their social capital quintile rankings between 2020 and 2024: those that remained in or moved to higher quintiles (i.e., high or increasing social capital) versus those that remained in or shifted to lower quintiles (i.e., low or decreased social capital). Aggregate neighborhood-level measures of perceptions regarding the pandemic’s impact on life circumstances and attitudes toward preventive measures will be based on aggregated individual survey responses. Logistic regression will be used to examine the association between patterns of change in neighborhood social capital and various outcomes related to perception and attitudes, adjusting for neighborhood-level sociodemographic and economic characteristics.

2 Quantitative sub-study 2: cross-level interaction between individual and neighborhood social capital and its effects on physical and mental health, attitudes toward, and adherence to preventive measures

This study will address our second specific aim: to analyze the effects of individual social capital on physical and mental health, as well as attitudes toward and adherence to preventive measures for different population sub-groups, living in neighborhoods with different social capital profiles. Specifically, this sub-study will examine; (i) If changes in access to individual social capital is associated with self-rated physical and mental health, and with attitudes toward and adherence to preventive measures; (ii) If individual social capital is associated with subsequent physical and mental health diagnoses, hospitalizations and medications, and if these associations are moderated by gendered, and (iii) If neighborhood social capital moderate the association between individual social capital and the health outcomes outline above. This sub-study will be based on panel data comprising individuals who responded to the social capital surveys both in 2020 and at follow-up in 2024. The final sample consists of 3,508 individuals, representing 70.9% of the 4,947 individuals who participated in the 2020 survey.

The primary variables of interest are various dimensions of individual social capital, operationalized as follows:

- *Bonding social capital*—access to social networks involving family, friends and neighbors characterized by reciprocal help and support. We asked four types of questions related to these groups of individuals: (i) whether an individual considered each group as part of their social networks, measured as a qualitative variable with dichotomous categories of yes and no; (ii) the frequency of real-life interaction with each group, measured as a quantitative variable with a Likert scale ranging from never to every day; (iii) whether an individual could receive help or practical support from each group, measured as a qualitative variable with dichotomous categories of yes and no, and; (iv) whether an individual had someone with whom he/she could share his/her innermost feelings, measured as a qualitative variable with dichotomous categories of yes and no. In generating these offline bonding social capital indices, we will conduct a factor analysis of mixed data using the FAMD package in R. FAMD is a principal component method for exploring data with continuous and categorical variables. We will use the missMDA package in R to impute missing individual social capital data. FAMD results in a factor score, which will later be grouped to represent a range from individuals with low bonding social capital (lower score) to those with high bonding social capital (higher score). In the further analyses, we will control for online bonding social capital (i.e., the frequency of online interactions with the same groups).
- *Bridging social capital*—access to broader and more diverse social networks, including contact with individuals of different ethnic backgrounds, participations in civic associations and attendance at public events. A summary measure using Principal Component Analysis (PCA) will be constructed based on the following question: whether an individual has; (i) been engaged in at least one out of 11 listed associations during the last 12 months (yes/no); (ii) participated in public events during the last 12 months (yes/no); (iii) an offline social network consisting of more than 15 people (yes/no); (iv) an online social network consisting of more than 50 people (yes/no); (v) at least one person with other ethnic background in their offline social networks (yes/no); (vi) more than 15 people with other ethnic background in their online social networks (yes/no).
- *Trust* will be measured across three dimensions: (i) generalized trust, assessed by the question “Do you generally think that people can be trusted, even those you are not personally acquainted with?” (yes/no); (ii) personalized trust, assessed by the question “Do you feel that you can trust people in the area where you live?” (yes/no); and (iii) institutional trust, defined as having very or rather high trust in at least eight out of 16 listed public institutions.

Self-rated physical and mental health will be measured using the RAND-36 survey tool (Swedish version). The survey data will be linked with administrative register data to obtain detailed demographic and socioeconomic data, including education, occupation, income, country of birth, and marital status (sourced from Statistics Sweden’s Swedish Longitudinal Integrated Database for Health Insurance and Labor Market Studies—LISA database). Health outcomes such as cardiovascular-, obesity- related, and mental health diagnoses, as well as hospitalizations and prescribed medications, will be retrieved from the National Patient Register and the National Prescribed Drug Register. Data on COVID-19 vaccination will be obtained from National Vaccination Register. Previous research suggests that the prevalence of mental illness, cardiovascular diseases, and obesity increased during the COVID-19 pandemic (44, 45). Furthermore, these health outcomes have been found to be associated with levels of social capital (46).

We will employ a linear mixed-effect model to examine how changes in various dimensions of individual social capital—bonding and bridging social capital, and trust between 2020 and 2024 are associated with various outcomes at follow-up. These outcomes include self-rated health, physical and mental health, attitudes toward and adherence to preventive measures and COVID-19 vaccination. We will conduct multilevel logistic regression analyses for binary outcomes (self-rated health, attitudes, and adherence) and multilevel linear regression models for continuous outcomes (physical and mental health scores)). In all models, individuals (Level 1) will be nested within neighborhoods (Level 2). Further, we will employ a shared frailty model, an extension of the Cox proportional hazards model, to evaluate whether baseline individual social capital predicts incident cardiovascular, obesity-related, and mental health diagnoses, as well as related hospitalizations and medication use (47, 48). The shared frailty model introduces a random effect (frailty term) into the hazard function, allowing for unobserved heterogeneity across individuals who may share latent risk factors. In practice, this means that the hazard for each individual is multiplicatively scaled by an unobserved frailty term, which captures dependence within clusters and accounts for correlated survival times. This approach is relevant to this study, as individuals are embedded in neighborhoods and may be subject to unmeasured contextual influences. By explicitly modeling this heterogeneity, the frailty model provides more accurate estimates of the association between social capital and subsequent health outcomes, reducing the risk of biased hazard ratios due to unobserved confounding.

In addition, we will construct mixed-effects models with cross-level interactions between individual-level and neighborhood-level social capital. These models will assess whether neighborhood social capital moderates the relationship between individual social capital and the health outcomes described above. The multilevel models will adjust for a predefined set of covariates. At the individual level, these include age, gender, marital status, education, employment status, immigrant background and prior comorbidities. At the neighborhood level, contextual covariates include mean income, and proportion of households with: higher education, pensioners, children under 12, welfare-benefits, unemployment, and single-parents. To assess potential effect modification, we will test theoretically relevant interactions, such as gender and social capital, by including cross-product terms in the models. Statistically significant or theoretically important interactions will be retained, while main-effects models will be presented otherwise.

3 Qualitative sub-study 3—exploring how the pandemic and the preventive measures impacted people’s access to and utilization of individual social capital

This qualitative sub-study will be based on interviews with participants who took part in both surveys in 2020 and 2024 and addresses the third specific objective, i.e., *to analyze how the pandemic and its associated preventive measures impacted people’s access to and utilization of individual social capital.* Analyses of quantitative survey data from this cohort will enable us to examine whether access to social capital increased or decreased during the pandemic (i.e., from 2020 to 2024) and whether these patterns differ for different social groups. This qualitative study will provide a deeper understanding of the patterns identified in the quantitative survey. The questions explored in this study include: What processes and conditions could explain whether individual social capital can be mobilized (i.e., increased) or diminished during a social crisis? In what ways and under what conditions could existing social capital be utilized during a pandemic, and how did individual’s social capital change or transform into new forms during this period? Did the significance of online and geographically close networks (e.g., in the neighborhood) change during the pandemic?

We will invite a purposive sample of participants from the cohort who responded to both the 2020 and 2024 surveys and who indicated their willingness to participate in follow-up interviews by providing their contact information. Based on their survey responses, we will identify three groups: (i) individuals who experienced an increase in bonding social capital, (ii) those who experienced a decrease, and (iii) those with relatively stable (indifferent) levels of bonding social capital over the 4-year period. Individual bonding social capital will be measured using individual factor scores (as described above) for 2020 and 2024. These scores will be divided into 10 groups, ranging from 1 (lowest) to 10 (highest), with each participant assigned a score for both years. A change of more than three steps between 2020 and 2024 will be classified as an increase or decrease, while a change of three steps or fewer will be considered indifferent. Based on this classification, three lists of participants will be created. From the increase and decrease groups, individuals will be randomly selected using a random number generator and invited to participate in follow-up interviews. Thus, the preceding quantitative survey will serve as the sampling frame for this qualitative sub-study, enabling systematic case comparison (49).

Participants will be invited via telephone, and interviews will preferably be conducted face to face at a location convenient for the participant. Prior to the interviews, we will prepare a summary of each participant’s survey responses from 2020 to 2024, which will serve as a basis for the qualitative interview. Their responses to questions about individual social capital, i.e., interactions and reciprocal help and support within their social networks of family, friends, and neighbors (bonding social capital), as well as size and composition of their social networks, involvement in civic associations, and participation in public events (bridging social capital) generalized, personalized and institutionalized trust (to people in general, neighbors and authorities), will be summarized and compared across the two survey years.

The interviews will be conducted as conversations around these survey responses, aiming to explore participants’ reflections on potential changes over time. The interview will begin by revisiting the participant’s life situation in 2020, using their survey responses as a starting point. The conversation will then explore how the participant managed daily life during the pandemic, including aspects such as social activities and interactions with friends and family, shopping habits, work or school routines, travel, access to information and support, and how any feelings of worry were addressed. Comparing responses to the same questions from 2020 to 2024 will help participants recall past experiences more accurately and reduce the risk of recall bias. Using participant’s repeated survey responses as the basis for follow-up interviews offers an innovative approach to data triangulation. This could deepen the understanding of the survey findings by adding personal stories behind increases or decreases in social capital during the pandemic. This strategy aligns with a mixed method-sequential design, where the results from the first (quantitative) phase inform the data collection of the subsequent (qualitative) phases (43). This sub-study will contribute to explaining how, and under what conditions, social capital may serve as a buffering resource during social crises.

Data analysis will follow a longitudinal trajectory analysis approach. While Grossoehme and Lipstein (50) describe trajectory analysis of longitudinal qualitative data, our study combines both quantitative and qualitative data. Participants’ responses to the survey questions in 2020 and 2024, along with quantitative analyses of potential changes over time, will be qualitatively explored and analyzed through the follow-up interviews. This approach enables a joint analysis of participants’ views and reflections on how the pandemic impacted their social capital, providing a complementary perspective to the overall patterns found in the survey data. Interview data will be coded following Grounded Theory methodology, using initial, selective, and theoretical coding (51). All interviews will be recorded and transcribed word by word, by means of the digital transcription tool Klang.ai. The coding procedure will be facilitated by the freely available software OpenCode. First, an initial open coding of all transcripts will be conducted, line by line, without having specific theoretical ideas in mind. In the next selective coding phase, our initial codes will be reviewed, sorted, and grouped into preliminary categories in accordance with their content. This procedure will reveal what codes and categories are most relevant for the purpose of our study. In the theoretical coding phase, the most relevant categories and their links will be theorized to come up with an abstract level of understanding of our research questions.

Data from the two groups—those who experienced a decrease versus an increase in social capital—will be treated as two separate datasets to facilitate comparisons between participants who reported different trajectories of social capital during the pandemic. We anticipate interviewing approximately 10–12 individuals who experienced an increase in social capital and a similar number who experienced a decrease, for a total of approximately 20–24 participants.

**Figure 1:**
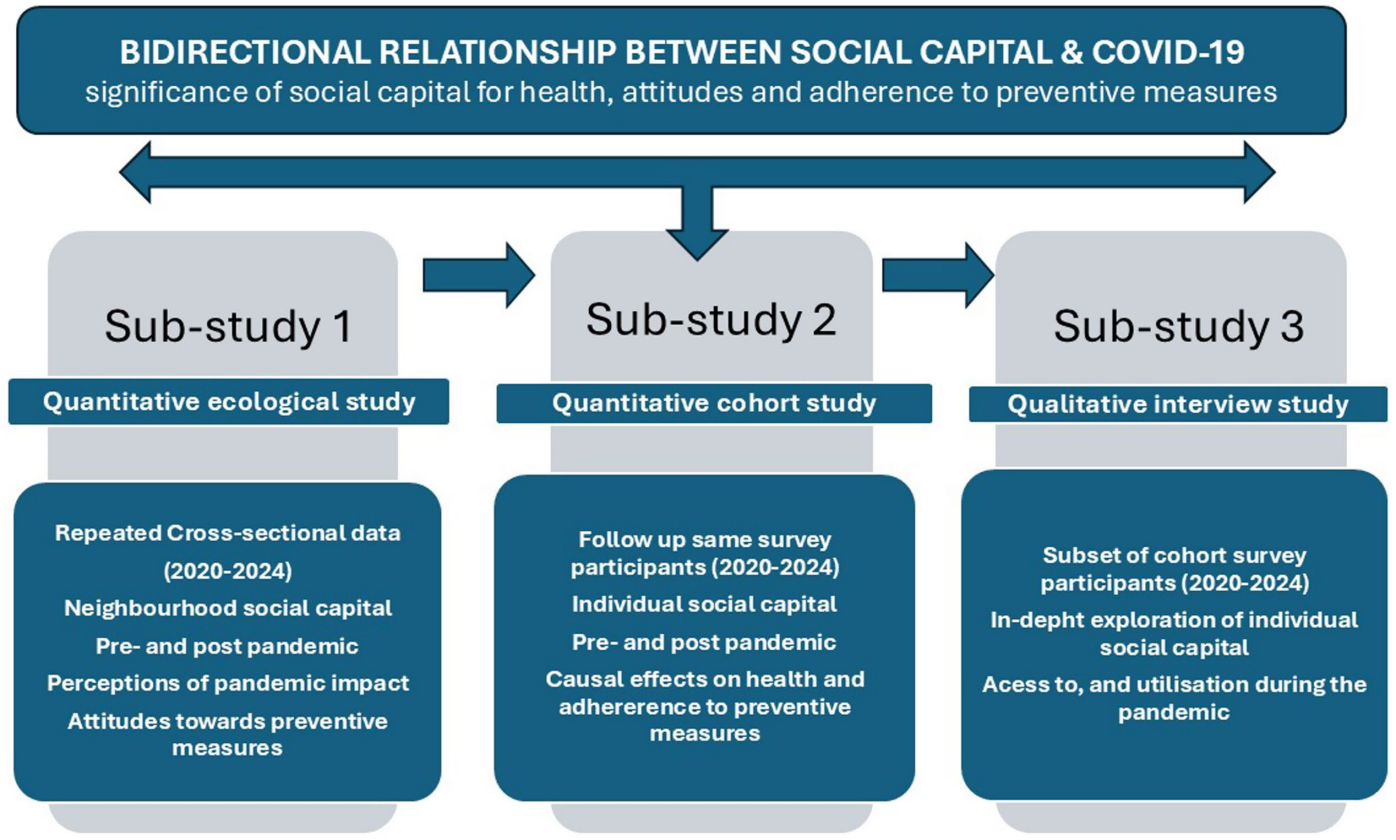
Illustration of the three sub-studies.

The three sub-studies and how they build on each other to fulfill the overall aim of the project are illustrated in Figure 1 below.

## Discussion

### From research to practice

Rapid societal changes and social crises imply new challenges for individuals and society, not least linked to resilience and crisis management and how to ensure socially sustainable and health-promoting living environments for all. Increasing the capacity of individuals and society to deal with new threats is crucial, as is knowledge of how the unequal health consequences of societal crises can be counteracted. Social capital is appealing as a potential buffering resource for individuals and for enhancing community resilience. Knowledge about the complex links between social capital and COVID-19 outcomes, i.e., how the pandemic affected access to social capital and how social capital influenced health outcomes and crisis management, could thus be essential for understanding and counteracting unequal health consequences of social crises. However, social capital is dynamic and may also change due to crises. Hence, the associations between social capital and various outcomes are complex. For the project results to be relevant and implementable, they must be generated in close collaboration between research and practice.

This project is carried out in Umeå Municipality, where extensive research on social capital, health and social sustainability has been conducted since 2006 through a long-term research-to- practice collaboration. Our previous results are currently being implemented in the strategic municipal plan for developing socially sustainable and health-promotive neighborhoods. The research team has also been involved in district dialogs pioneered by the Umeå Municipality and contributed to knowledge on the significance and development of social capital in the municipality. This research-to-practice collaboration has enabled Umeå Municipality to utilize our scientific mapping of social capital in designing urban social sustainable development. This proposed study contributes novel and unique knowledge about how a social crisis affects unequal living conditions between groups and geographical areas and can thus provide a basis for what actions are needed to reduce the adverse health consequences of social crises. This knowledge is essential for national and local policy and planning for crisis preparedness, public health, and social sustainable development.

Our research- to-practice collaboration is characterized by mutual respect for the complementary roles of research and practice. From the research perspective, Umeå Municipality is an interesting case for exploring research questions on contemporary societal issues. In addition, our collaboration with the municipality facilitates and ensures the spread and applicability of the results. From the municipal perspective, collaboration with research offers a knowledge base for decision-making and ensures that critical scientific perspectives are considered in practice.

### Considerations of social and gender inequalities

Many social and gender inequality perspectives need to be considered in this project. Previous research indicates that the protective effects of social capital differ for population sub-groups defined by, e.g., gender, ethnicity, and age groups (35). Access to social capital is also generally higher among people with higher socioeconomic positions (37). Further, gender inequality in the gains and costs of social capital has been found in that women are expected to be the primary provider of social support, not least within family networks (52). This gendered pattern could potentially have been reinforced during the pandemic crisis. The COVID-19 pandemic also revealed inequalities with older people, men, individuals with lower socioeconomic status, and individuals born in certain countries, and some residential areas have been hit harder than others (53, 54). Less advantaged groups are also less likely to benefit from social interactions through Internet communication at the restricted time during the pandemic, making them even more vulnerable (55). Further, women, who are generally more prone to mental health burdens, experienced more mental health effects related to COVID-19 and implemented preventive measures (56). Our multidisciplinary mixed-method study will contribute to further knowledge about the complex links between gender, socioeconomic position, social capital, and physical and mental health.

## Ethics and dissemination

Questions regarding health, social networks, attitudes, and adherence to preventive measures may be perceived as sensitive. Nevertheless, we assess the risks to research participants as low. None of the data collection methods pose any risk of immediate harm. Participation in both surveys and interviews is entirely voluntary and based on informed consent. The procedures for data collection, informed consent, data management, processing, and handling have been approved by the Swedish Ethical Authority (Dnr 2023-05584-01). All data will be treated confidentially. Register data will be anonymized, and the relevant authorities will facilitate the linkage to survey data. Results will be presented only at aggregated level. Participation in interviews is voluntary, and participants will receive comprehensive written and oral information about how their data will be used and handled.

The project will follow the fundamental ethical principles for research involving human participants. *The autonomy princip*le: We will ensure that potential participants receive sufficient information and time to understand it before making an independent decision about participation. The participants have the right to withdraw from the study at any time without providing a reason. *The non-maleficence principle:* None of our data collection methods pose any risk of harm to participants. We will inform all participants that their information will be treated confidentially and only be presented at an aggregated level. *The beneficence principle:* The overall aim of the study is to contribute knowledge that can enhance society’s crisis preparedness. Additionally, being invited to share their experiences and contribute to research may offer some participants a sense of personal benefit.

*The justice principle:* We will ensure that participants are treated fairly, and that the selection of participants is based on relevant research considerations, not on convenience, bias, or discrimination. Efforts will be made to include individuals from diverse backgrounds to ensure that the benefits and burdens of research participation are equitably distributed.

Beyond scientific publications, an important communication channel for our research findings will be our ongoing collaboration with Umeå Municipality and other municipalities. Northern Sweden is experiencing rapid societal transformation with growing industries and societies. This transformation raises important issues on how to grow and, at the same time, preserve health and social sustainability on equal terms. Our longitudinal research-to- practice collaboration in Umeå Municipality about social capital, health and social sustainability has already gained interest among other municipalities in Northern Sweden. We foresee several opportunities (e.g., the National Safety Conference), where the results of this proposed project can be communicated with representatives for municipalities that currently undergo pervasive societal transformations.
